# One-Pot Synthesis of Hyperbranched Polyurethane-Triazoles with Controlled Structural, Molecular Weight and Hydrodynamic Characteristics

**DOI:** 10.3390/polym14214514

**Published:** 2022-10-25

**Authors:** Sergei V. Karpov, Artem Iakunkov, Alexander V. Akkuratov, Artem O. Petrov, Eugenia O. Perepelitsina, Georgiy V. Malkov, Elmira R. Badamshina

**Affiliations:** 1 Department of Polymers and Composite Materials, Federal Research Center of Problems of Chemical Physics and Medicinal Chemistry of Russian Academy of Sciences, 142432 Chernogolovka, Russia; 2 Department of Fibre and Polymer Technology, KTH Royal Institute of Technology, SE-100 44 Stockholm, Sweden

**Keywords:** hyperbranched polymers, polyurethane-triazoles, degree of branching, polyaddition

## Abstract

We report a simple and convenient approach to the one-pot synthesis of hyperbranched polyurethane-triazoles with desirable properties. This method is based on in situ generation of an *AB*_2_ + *A*_2_ + *B*_4_ azide-acetylene monomer mixture of known composition, due to quantitative reactions of urethane formation between isophorone diisocyanate (IPDI), 1,3-diazidopropanol-2 (DAPOL) (in the first stage) and propargyl alcohol (in the second stage). The obtained monomer mixture can be involved in step-growth polymerization by azide-alkyne cycloaddition without additional purification (in the third stage). The properties of the resulting polymers should depend on the composition of the monomer mixture. Therefore, first the model revealing the correlation between the monomer composition and the ratio and reactivity of the IPDI and DAPOL active groups is developed and proven. In addition, the newly developed structural kinetic model considering the substitution effect at polyaddition of the complex mixture of monomers allows the prediction of the degree of branching of the target polymer. Based on our calculations, the hyperbranched polyurethane-triazoles were synthesized under found conditions. All products were characterized by ^1^H NMR, FTIR, SEC, DLS, DSC, TGA and viscometry methods. It was shown that the degree of branching, molecular weight, intrinsic viscosity, and hydrodynamic radius of the final hyperbranched polymers can be specified at the first stage of one-pot synthesis. The obtained hyperbranched polyurethane-triazoles showed a degree of branching from 0.21 to 0.44 (calculated DB-0.25 and 0.45, respectively).

## 1. Introduction

The synthesis and investigation of new functional materials, such as highly and hyperbranched polymers has attracted considerable attention in the last few years [1,2,3,4]. Hyperbranched polymers with many functional end-groups are considered as promising materials for design of ecological flame-retardant polyurethanes [5], new catalytic systems [6], biomaterials [7], and other applications [8,9,10]. They exhibit high solubility in common organic solvents and high thermodynamic compatibility with other polymers, low viscosity, and high sorption capacity.

Hyperbranched polymers can be prepared via the three-dimensional step-growth polymerization of *AB_n_*-type monomers (*n* ≥ 2), where A and B groups can only react with each other. This is the simplest example of a topological mechanism which enables the formation of hyperbranched polymers [11]. One of the main advantages of step-growth polymerization of *AB_n_*-type monomers is the absence of cross-linking (gelation), even at full conversion of *A* groups [12]. However, the preparation of these monomers often involves multi-step and complex reactions. Furthermore, the isolation of products is challenging, due to highly reactive groups [13]. Another way to obtain highly and hyperbranched polymers is the co-polymerization of symmetric (*A*_2_ + *B*_3_, *A*_2_ + *B*_4_, *A*_3_ + *B*_3_ et al.) [14,15,16] and asymmetric monomer couples (*A*_2_ + *CB*_2_, *A*_2_ + *B*_2_*B*′, *AA*′ + *B*_3_, *AC* + *B*_3_ et al.) [17,18]. These approaches are limited by the fact that branched polymers can be synthesized near the critical conversion of gelation, or where there is a significant difference in the reactivity of functional groups. In addition, there are strong limitations in the choice of starting monomer couples for the synthesis of branched polymers with such an unusual structure as triazoles. Thus, it is necessary to develop novel approaches to the synthesis of highly and hyperbranched polymers by the simple step-growth polymerization of *AB*_2_ type monomers, using a wide range of available reagents.

Previously, we developed an approach where the branched polyurethane-triazoles are formed after the synthesis of the *AB*_2_ + *A*_2_ + *B*_4_ monomer mixture [19]. The synthesis procedure involves two quantitative reactions: urethane formation and azide-alkyne cycloaddition (AAC). The first step is forming a diazide-isocyanate precursor, through a reaction between 1,3-diazidopropanol-2 (DAPOL) and an excess of symmetric hexamethylene diisocyanate (HMDI), to reduce the amount of *B*_4_-type monomer. The diazide-acetylene (*AB*_2_-type monomer) can be obtained by reaction of the precursor with propargyl alcohol (PrAl) only after purification of diazide-isocyanate from HMDI, to protect the mixture against the formation of the inappropriate component *A*_2_-type monomer. Finally, the synthesis of branched polyurethane-triazoles can be carried out through the step-growth polymerization of the *AB*_2_ + *A*_2_ + *B*_4_ monomer mixture by the AAC reaction.

In this work, we suggest an approach to the controlled and targeted one-pot synthesis of hyperbranched azide-containing polyurethane-triazoles, based on asymmetrical isophorone diisocyanate (IPDI) with NCO-groups, which exhibited strongly different reaction activity. Recently, we have reported that the ratio of reaction rate constants for the cycloaliphatic NCO-group (*k_cal_*) and aliphatic NCO-group (*k_al_*) of IPDI approaches 40 when it reacts with DAPOL [20,21]. Therefore, utilizing IPDI would allow an increase in the amount of *AB*_2_-type monomer in the generated mixture, without additional isolation and purification.

## 2. Materials and Methods

### 2.1. Materials

Distillation of IPDI was performed under reduced pressure (~3 Pa) at 60 °C; the isocyanate content was analyzed using a previously reported method [22], and it was 99.9%. PrAl and catalyst dibutyltin dilaurate (DBTDL) (≥98.0% pure, all from Sigma Aldrich, Geel, Belgium) were used without further purification. DAPOL was synthesized according to methods from [23]; the purity was ≥98.0%, as was shown using FTIR, ^1^H NMR, and elemental analysis. Solvents were purified by standard procedures [24].

### 2.2. Characterization

IR spectra were recorded on a Bruker Alpha FTIR spectrometer (Bruker, Ettlingen, Germany). Solid samples were analyzed using the ATR module.

The ^1^H NMR spectra were obtained using a Bruker AVANCE III BIOSPIN spectrometer (500 MHz, Bruker, Ettlingen, Germany) with DMSO-*d*_6_ and TMS as an internal standard.

The molecular weight distribution was analyzed using size-exclusion chromatography (SEC), using a Waters GPCV 2000 chromatograph (column PL-gel, 5 µm, MIXED-C, 300 × 7.5 mm, Waters Corporation, Milford, MA, USA) equipped with refractometer, viscometer and light-scattering detector WYATT DAWN HELEOS II (λ = 658 nm). N-methylpyrrolidone with a small amount of LiCl was used as an eluent. All measurements were carried out at 70 °C; flow rate was 1 mL/min. “EMPOWER”, and Astra 5.3.2.20 software (Wyatt Technology, Santa Barbara, CA, USA) was used for data processing. The absolute parameters of the molecular weight distribution of hyperbranched polymers were calculated, based on light-scattering detector data.

Thermal analysis was performed using a differential scanning calorimeter Mettler Toledo DSC822e in the temperature range from −70 to 170 °C in an inert atmosphere, with a heating rate of 5 °C/min. The glass transition temperature of polymers (T_g_) was found from the temperature relationship W = f(T). Thermo-gravimetric analysis (TGA) was carried out using a Mettler-Toledo TGA/SDTA 851e/SF/1100 from 25 to 350 °C, in an inert atmosphere (Ar, 20 mL/min), with a heating rate of 10 °C/min and 2 °C/min in ceramic crucibles. The decomposition temperature (T_d_) was found, using a minimum of the first derivative from weight loss vs. temperature.

The solutions of polyurethane-triazoles in N-methylpyrrolidone with a small amount of LiCl were analyzed with the dynamic light scattering method using a Photocor Compact (Photocor Instruments Inc., College Park, MD, USA, detection angle 90°, 654 nm wavelength laser diode, 70 °C). The solutions were preliminarily filtered through a PES membrane with a 0.45 μm pore diameter, and kept for 1 h at a constant temperature before measurement. The data processing was performed using DynaLS v. 2.8.3 software (Alango, Israel) by the regulation method (Distribution Analysis). The Einstein-Stokes equation was used to find the value of the hydrodynamic radius of dispersed particles (Rh).

Rheological characteristics were analyzed using the Ubbelohde viscometer at 70 °C for polymer solutions in N-methylpyrrolidone.

WATERS chromatograph, Symmetry 300 C18, 5 µm, 4.6 × 250 mm column, methanol/water eluent (75/25 by volume), 0.7 mL/min flow rate, UV-detectors with diode array PDA 996 (210 nm–400 nm), and WATERS 2414 refractometric detector were used for reversible-phase chromatography (RPC) analysis. RPC-chromatograms registration and data processing were carried out using the “EMPOWER” software package.

The resulting mixture’s composition was identified using the RPC method for the reactions between IPDI and DAPOL interaction in CCl_4_ with a variation of [NCO]_0_/[OH]_0_ ratios. After reaction, the mixture of products was treated with methanol (indicated by FTIR spectroscopy) to deactivate the NCO groups. The methanol/water eluent (75/25 by volume) was efficient for separating the reaction mixture during RCP characterization.

### 2.3. One-Pot Synthesis of Hyperbranched Polyurethane-Triazoles

The synthesis of the monomer mixture for hyperbranched polyurethane-triazoles was performed in situ in CCl_4_. The first step was forming a diazide-isocyanate precursor through a reaction between DAPOL and IPDI. A 50 mL flask was filled with 25 mL of CCl_4_ and calculated amounts of IPDI and DAPOL under argon in ratio [NCO]_0_/[OH]_0_ = 1.50–1.93, [OH] = 0.5 mol/L. The reaction mixture was stirred at 20 °C with a DBTDL catalyst (concentration of 5 mmol/L) for 6 h. FTIR spectroscopy was used for monitoring the reaction by decreasing the intensity of NCO-groups IPDI valent oscillations (2270 cm^−1^, the molar extinction coefficient of the NCO-groups, is equal to 1076 L/(mol·cm) in CCl_4_).

At the end of the precursor synthesis, the *AB*_2_ + *A*_2_ + *B*_4_ monomers mixture was obtained through a one-pot reaction of the leftover NCO groups of the precursor with a calculated amount of PrAl. FTIR spectroscopy was used for monitoring the end of the reaction by the disappearance of the NCO groups absorption band. The obtained monomer mixture was dried in a vacuum (10^−2^ torr) at room temperature. In addition, the completion of the reaction was proven by ^1^H NMR, where signals for 1,4- and 1,5-disubstituted-1,2,3-triazoles were observed. The conversion of ethynyl groups did not exceed 10% (see Figure S1 in Supporting Information).

NMR data of the monomer mixture was obtained in the reaction of DAPOL with IPDI at ratio [NCO]_0_/[OH]_0_ = 1.70: ^1^H NMR, ppm: δ = 8.05 (s, H(12′ ^1,4^), 7.70 (s, H(12′ ^1,5^), 7.51–6.82 (m, H(3), H(9)), 5.23–5.0 (m, H(11′), H(13′ ^1,4^), H(13′ ^1,5^)), 5.0–4.78 (m, H(11)), 4.71–4.46 (m, H(13), H(10′ ^1,4^), H(10′ ^1,5^)), 3.75–3.42 (m, H(5), H(10), H(12)), 2.88–2.58 (m, H(8)), 1.77–1.03 (m, H(2), H(4), H(7)), 1.03–0.70 (m, H(1), H(6)). FTIR, cm^−1^ (capillary film): 3446, 3342 (NH), 2985, 2957, 2926, 2873, 2874 (CH_2_), 2106 (N_3_), 1730 (C=O), 1514 (NH).

**Figure 1:**
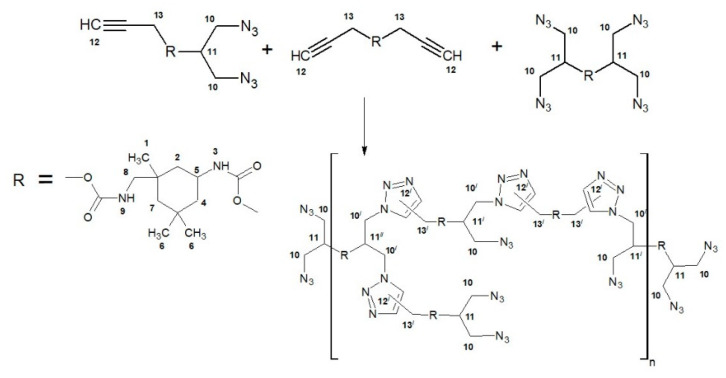
Scheme for the synthesis of hyperbranched polyurethane-triazoles.

Finally, the synthesis of hyperbranched polyurethane-triazoles was carried out through the step-growth polymerization of the *AB*_2_ + *A*_2_ + *B*_4_ monomer mixture by the AAC reaction in bulk at T = 100 °C; see Figure 1. The signal intensity ratio of asymmetric valent oscillations of azide groups and valent oscillations of carbonyl groups of urethane in the FTIR spectra (at ca. 2100 cm^−1^ and 1705 cm^−1^, respectively) allowed the control of the degree of polyaddition reaction.

NMR data for hyperbranched polyurethane-triazoles obtained at ratio [NCO]_0_/[OH]_0_ = 1.70: ^1^H NMR, ppm: δ = 8.05 (s, H(12′ ^1,4^), 7.70 (s, H(12′ ^1,5^), 7.51–6.82 (m, H(3), H(9)), 5.50–5.30 (m, H(11″)), 5.29–4.97 (m, H(11′), H(13′ ^1,4^), H(13′ ^1,5^)), 4.97–4.83 (m, H(11)), 4.71–4.51 (m, H(10′ ^1,4^), H(10′ ^1,5^)), 3.75–3.42 (m, H(5), H(10)), 2.88–2.58 (m, H(8)), 1.77–1.03 (m, H(2), H(4), H(7)), 1.03–0.70 (m, H(1), H(6)). FTIR ATR, cm^−1^: 3327 (NH); 2952, 2926, 2869, 2846 (CH_2_); 2100 (N_3_); 1705 (C=O); 1523 (NH); 1460 (triazole).

## 3. Results and Discussion

The composition of the monomer mixture in the reaction of IPDI with DAPOL can be controlled by varying the [NCO]_0_/[OH]_0_ ratio, due to difference in *k_cal_* and *k_al_*. When changing these parameters, the formation of mono-substituted (potential *AB*_2_ type monomer), disubstituted IPDI (*B*_4_ type monomer), and the diisocyanate derivative (*A*_2_ type monomer) is possible. Therefore, it is necessary to understand the composition of the resulting mixture of *AB*_2_ + *A*_2_ + *B*_4_ monomers. The mixture composition will also influence the properties of key products such as molecular weight distribution, rheological behavior, hydrodynamic characteristics, structural parameters, heat resistance, and glass-transition temperature (T_g_).

Thus, the prediction of the composition of the monomer mixture (*AB*_2_ + *A*_2_ + *B*_4_) at known [NCO]_0_/[OH]_0_ and *k_cal_*/*k_al_* = 40 is of great importance for the final step of polymer synthesis.

### 3.1. Influence of [NCO]_0_/[OH]_0_ Ratio on the Composition of AB_2_ + A_2_ + B_4_ Monomer Mixture and Structural-Kinetic Model of Their Polyaddition

**Figure 2:**
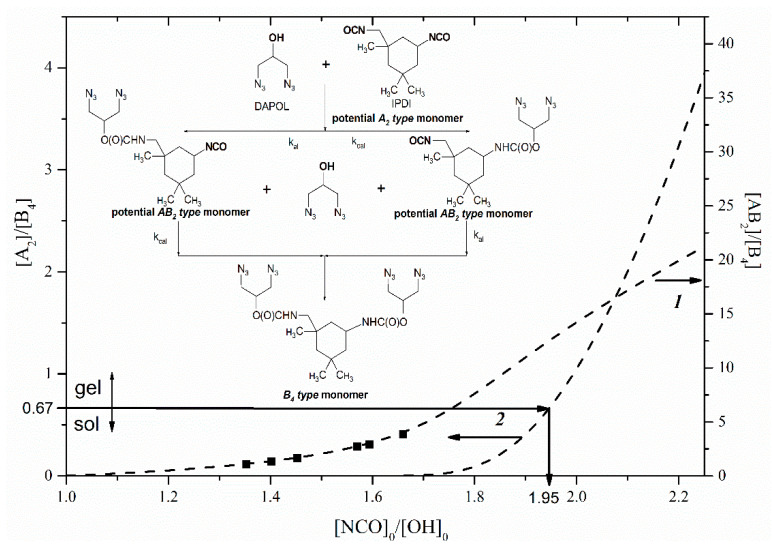
The simulated correlations (dashed line) between molar ratio of [*AB*_2_]/[*B*_4_] (right axis), [*A*_2_]/[*B*_4_] (left axis) and [NCO]_0_/[OH]_0_ in reaction IPDI with DAPOL and corresponding experimental data (solid squares).

The first synthetic step is the reaction of the IPDI-bearing aliphatic isocyanate group (rate constant *k_al_*) and cycloaliphatic isocyanate groups (rate constant *k_cal_*) with DAPOL (Figure 2). The obtained diazide-isocyanate precursor can act further as an *AB*_2_-type monomer. The unreacted IPDI can act further as an *A*_2_-type monomer. Further interaction of the precursor with DAPOL leads to the formation of a *B*_4_-type monomer. All components in the *AB*_2_ + *A*_2_ + *B*_4_ mixture are important for forming hyperbranched polyurethane-triazoles. In particular, the *AB*_2_-type monomer can be considered as a branching agent. The *A*_2_-type monomer can be considered as a cross-linking agent. Finally, the *B*_4_-type monomer decreases the molecular weight of the final polymers. The concentration of these compounds can be controlled by a different ratio [NCO]_0_/[OH]_0_.

In order to avoid gelation, we have to choose the concentration ratio of *A*_2_- and *B*_4_-type monomers. As was reported previously, gelation can occur at critical ratio [*A*_2_]/[*B*_4_] of 0.67 [25]. The composition of the product in reaction IPDI with DAPOL was simulated vs. [NCO]_0_/[OH]_0_ ratios, using differential equations as follows:

(1) $$\frac{d\left[\mathrm{IPDI}\right]}{\mathrm{dt}}=-k_{al}\;\left[\mathrm{IPDI}\right]\left[\mathrm{DAPOL}\right]-k_{cal}\left[\mathrm{IPDI}\right]\left[\mathrm{DAPOL}\right]$$

(2) $$\frac{d\left[AB_{2}^{al}\right]}{\mathrm{dt}}=-k_{cal}\;\left[AB_{2}^{al}\right]\;\left[\mathrm{DAPOL}\right]+k_{al}\left[\mathrm{IPDI}\right]\left[\mathrm{DAPOL}\right]\;$$

(3) $$\frac{d\left[AB_{2}^{cal}\right]}{\mathrm{dt}}=-k_{cal}\;\left[AB_{2}^{cal}\right]\;\left[\mathrm{DAPOL}\right]+k_{al}\left[\mathrm{IPDI}\right]\left[\mathrm{DAPOL}\right]$$

(4) $$\frac{d\left[B_{4}\right]}{\mathrm{dt}}=k_{cal}\;\left[AB_{2}^{al}]\;\left[\mathrm{DAPOL}\right]+k_{cal}[AB_{2}^{al}\right]\left[\mathrm{DAPOL}\right]$$

**Figure 3:**
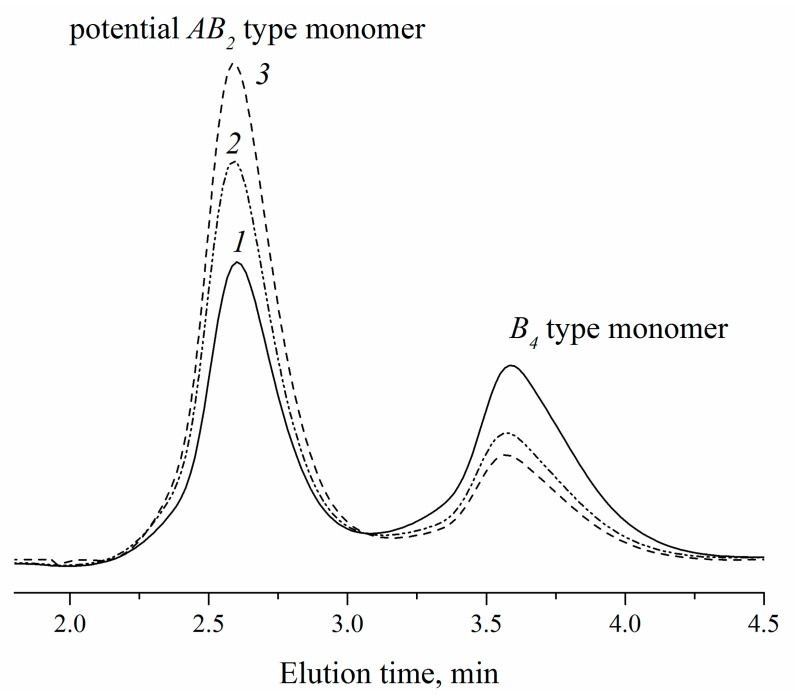
RPC for the products in reaction of IPDI with DAPOL, where the ratio [NCO]_0_/[OH]_0_ was 1.35 (1), 1.57 (2), and 1.66 (3).

The experimental correlations are based on RPC data for the products in reaction IPDI with DAPOL at a different [NCO]_0_/[OH]_0_ ratio (Figure 3). Obviously, with the increase of the [NCO]_0_/[OH]_0_ ratio, more amounts of the *AB*_2_-type monomer form in the mixture of *AB*_2_, *A*_2_ and *B*_4_. Only two peaks can be seen on RPC, which correspond to *AB*_2_- and *B*_4_-type monomers, while the formation of *A*_2_-type monomers is limited at selected [NCO]_0_/[OH]_0_ ratios (Figure 3). Attempts to achieve monomer separation with other ratios failed because of the limited solubility of the *A*_2_-type monomer. Nevertheless, there are enough experimental data to confirm a theoretical dependence between the composition of the *AB*_2_ + *A*_2_ + *B*_4_ monomer mixture and the [NCO]_0_/[OH]_0_ ratio in reaction IPDI with DAPOL. Based on this knowledge and the gelation condition ([*A*_2_]/[*B*_4_] ≥ 0.67), the ratio [NCO]_0_/[OH]_0_ ≤ 1.95 was found as acceptable for the synthesis of hyperbranched polyurethane-triazoles without the cross-linking process (Figure 2).

The composition of the *AB*_2_ + *A*_2_ + *B*_4_ monomer mixture is the first factor which influences the degree of branching (DB) of final polymers and their properties. However, the structure and properties of hyperbranched polyurethane-triazoles are also influenced by the kinetics of the polyaddition reaction. It is known that the substitution effect in the copper(I)-catalyzed azide-alkyne cycloaddition reaction for 1,3-diazides can change the rate constants (*k*_1_ and *k*_2_) for azide groups [26,27,28]. Since we use the same type of diazides, this effect should be also considered.

We proposed the structural-kinetic model of the general polyaddition reaction, considering the parameters found above.

It is well known [29] that the DB can be determined from the following relation:

(5) $$\mathrm{DB}=\frac{D+T}{D+T+L}\approx \frac{2D}{2D+L}\approx \frac{2T}{2D+L}$$

where D, L, and T are the number of branched, linear and terminal fragments of hyperbranched polymers.

**Figure 4:**
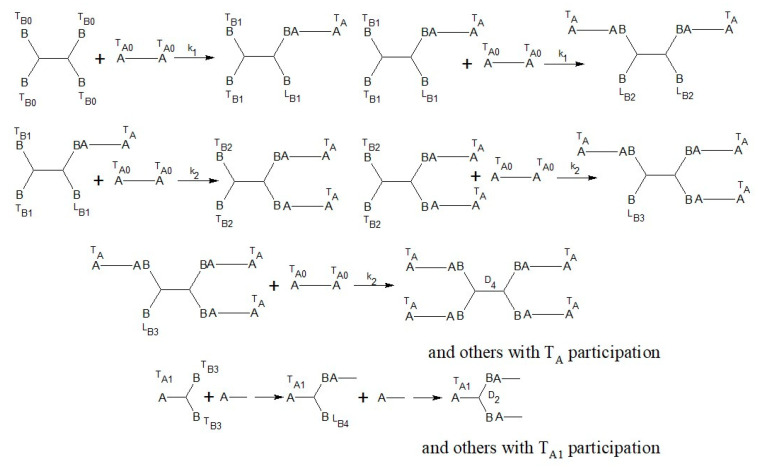
The elementary reactions for cycloaddition of *AB*_2_ + *A*_2_ + *B*_4_ monomer mixture with the substitution effect of B groups.

The kinetic-structural model for cycloaddition of the *AB*_2_ + *A*_2_ + *B*_4_ monomer mixture contains a set of elementary reactions (Figure 4).

The kinetic model of polyaddition can be described by a set of twenty-four reactions using 13 differential equations:

(6) $$\frac{d\left[T_{B0}\right]}{\mathrm{dt}}=-4k_{1}\;\left[T_{B0}\right]\left(\left[T_{A0}\right]+\left[T_{A}\right]+\left[T_{A1}\right]\right)$$

(7) $$\frac{d\left[T_{B1}\right]}{\mathrm{dt}}=\left(\left[T_{A0}\right]+\left[T_{A}\right]+\left[T_{A1}\right]\right)\left(-2k_{1}\;\left[T_{B1}\right]-2k_{2}\;\left[L_{B1}\right]+2k_{1}\;\left[T_{B0}\right]\right)$$

(8) $$\frac{d\left[T_{B2}\right]}{\mathrm{dt}}=\left(\left[T_{A0}\right]+\left[T_{A}\right]+\left[T_{A1}\right]\right)\left(-2k_{1}\;\left[T_{B2}\right]+2k_{2}\;\left[L_{B1}\right]\right)$$

(9) $$\frac{d\left[T_{B3}\right]}{\mathrm{dt}}=-2k_{1}\;\left[T_{B3}\right]\left(\left[T_{A0}\right]+\left[T_{A}\right]+\left[T_{A1}\right]\right)$$

(10) $$\frac{d\left[L_{B1}\right]}{\mathrm{dt}}=\left(\left[T_{A0}\right]+\left[T_{A}\right]+\left[T_{A1}\right]\right)\left(-k_{2}\;\left[L_{B1}\right]-k_{1}\;\left[T_{B1}\right]+k_{1}\;\left[T_{B0}\right]\right)$$

(11) $$\frac{d\left[L_{B2}\right]}{\mathrm{dt}}=\left(\left[T_{A0}\right]+\left[T_{A}\right]+\left[T_{A1}\right]\right)\left(-2k_{2}\;\left[L_{B2}\right]+k_{1}\;\left[T_{B1}\right]\right)$$

(12) $$\frac{d\left[L_{B3}\right]}{\mathrm{dt}}=\left(\left[T_{A0}\right]+\left[T_{A}\right]+\left[T_{A1}\right]\right)\left(-k_{2}\;\left[L_{B3}\right]+k_{1}\;\left[T_{B2}\right]+k_{2}\;\left[L_{B2}\right]\right)$$

(13) $$\frac{d\left[L_{B4}\right]}{\mathrm{dt}}=\left(\left[T_{A0}\right]+\left[T_{A}\right]+\left[T_{A1}\right]\right)\left(-k_{2}\;\left[L_{B4}\right]+k_{1}\;\left[T_{B3}\right]\right)$$

(14) $$\frac{d\left[T_{A0}\right]}{\mathrm{dt}}=\left[T_{A0}\right](-{2k}_{1}\left(\left[T_{B0}]+[T_{B1}]+[T_{B2}]+[T_{B3}\right]\right)-{2k}_{2}(\left[L_{B1}\right]+\left[L_{B2}\right]+\left[L_{B3}\right]+\left[L_{B4}\right]))$$

(15) $$\frac{d\left[T_{A}\right]}{\mathrm{dt}}=\left(\left[T_{B0}]+[T_{B1}]+[T_{B2}]+[T_{B3}\right]\right)\left(-k_{1}\left[T_{A}\right]+k_{1}\left[T_{A0}\right]\right)+(\left[L_{B1}\right]+\left[L_{B2}\right]+\left[L_{B3}]+[L_{B4}\right])\left(-k_{2}\left[T_{A}\right]+k_{2}\left[T_{A0}\right]\right)$$

(16) $$\frac{d\left[T_{A1}\right]}{\mathrm{dt}}=\left[T_{A1}\right]\left(-k_{1}\left(\left[T_{B0}\right]+\left[T_{B1}\right]+\left[T_{B2}\right]+\left[T_{B3}\right]\right)-k_{2}\left(\left[L_{B1}]+[L_{B2}]+[L_{B3}]+[L_{B4}\right]\right)\right)$$

(17) $$\frac{d\left[D_{2}\right]}{\mathrm{dt}}=k_{2}\left[L_{B4}\right]\left(\left[T_{A0}]+[T_{A}]+[T_{A1}\right]\right)$$

(18) $$\frac{d\left[D_{4}\right]}{\mathrm{dt}}=k_{2}\left[L_{B3}\right]\left(\left[T_{A0}]+[T_{A}]+[T_{A1}\right]\right)$$

This system of differential equations was solved using standard computational methods. As result, D, L, and T components can be expressed as follows:

(19) $$D=L_{B3}+2D_{4}+D_{2}+0.5{\;T}_{B2}$$

(20) $$L=L_{B1}+L_{B2}+L_{B3}+{\;L}_{B4}$$

(21) $$T=T_{B0}+T_{B1}+T_{B2}+{\;T}_{B3}$$

**Figure 5:**
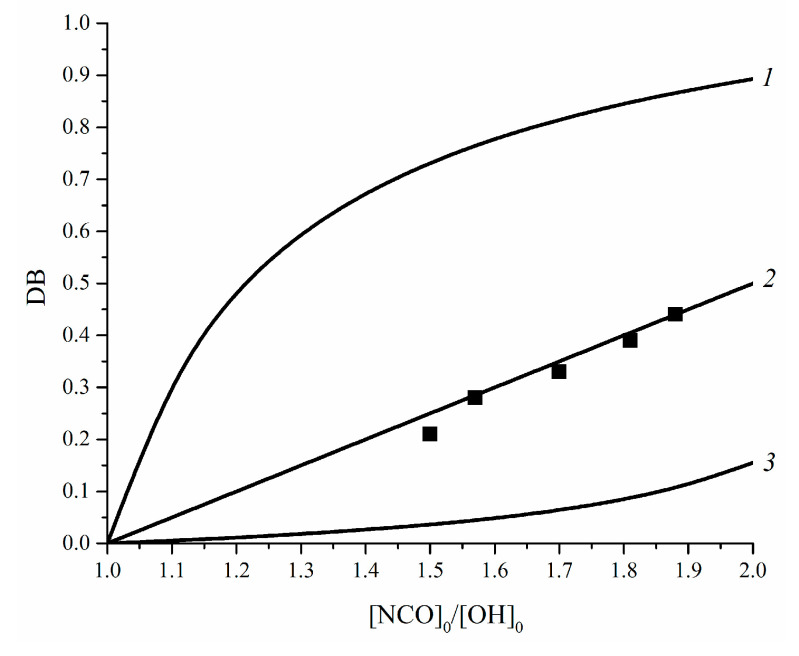
Correlation between DB_calc_ and initial ratio [NCO]_0_/[OH]_0_ for positive substitution effect (1), *k*_2_/*k*_1_ = 10; statistic polyaddition (2), *k*_2_/*k*_1_ = 1; negative substitution effect (3), *k*_2_/*k*_1_ = 0.1. Experimental data for synthesized polyurethane-triazoles are shown by solid squares.

This kinetic-structural model predicts a correlation between the initial ratios [NCO]_0_/[OH]_0_ for the reaction of IPDI with DAPOL and DB of the final hyperbranched polymers (Figure 5, 2). Moreover, this model allows the prediction of the influence of positive (Figure 5, 1) and negative (Figure 5, 3) substitution effects on DB.

As can be seen, DB decreases when the substitution effect is negative (*k*_2_/*k*_1_ < 1), and increases in the case of positive effect *k_2_/k*_1_ > 1. It is worth noting that these trends are true only for step-growth polymerization of the *AB*_2_ + *A*_2_ + *B*_4_ monomer mixture.

### 3.2. Synthesis of Hyperbranched Azide-Containing Polyurethane-Triazoles

**Figure 6:**
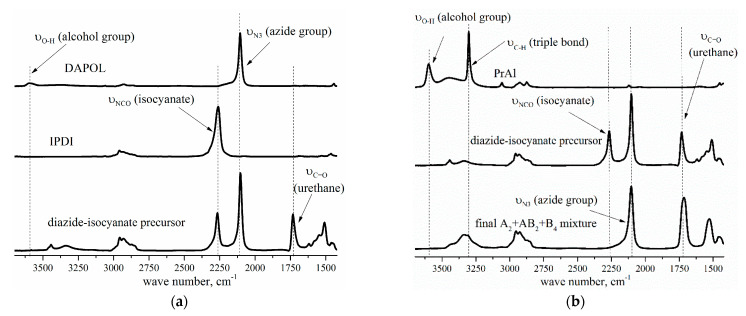
FTIR spectra of: (**a**) DAPOL, IPDI, the diazide-isocyanate precursor; (**b**) PrAl and *AB*_2_ + *A*_2_ + *B*_4_ monomer mixture. The ratio [NCO]_0_/[OH]_0_ was 1.7.

**Figure 7:**
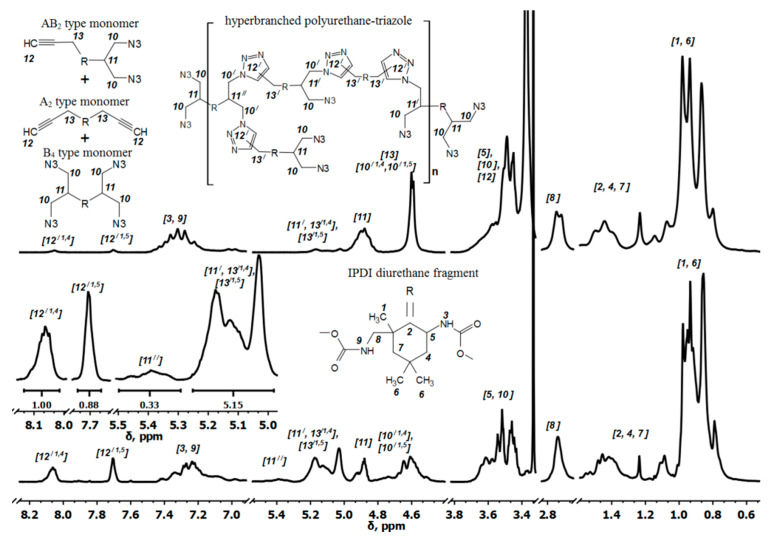
^1^H NMR spectra of *AB*_2_ + *A*_2_ + *B*_4_ monomer mixture (**top**) and hyperbranched polyurethane-triazoles (**bottom**) in DMSO-*d*_6_. The ratio [NCO]_0_/[OH]_0_ was 1.7.

In order to verify the proposed model, the two-step synthesis of hyperbranched polyurethane-triazoles was performed under the established conditions. First, the diazide-isocyanate precursor was obtained in the reaction of IPDI with DAPOL at [NCO]_0_/[OH]_0_ = 1.5 − 1.93. The reaction of the resulting precursor with PrAl at [NCO]/[OH] = 1 was then carried out. The composition of the monomer mixture was studied using FTIR (Figure 6) and ^1^H NMR spectroscopy (Figure 7).

In the spectrum of the diazide-isocyanate precursor (Figure 6a) no absorption band of the hydroxyl group from the DAPOL (υ -O-H at ca. 3600 cm^−1^) can be seen, while the band of the urethane group (υ -NHC(O)O- at ca. 1728 cm^−1^) is observed. The conversion of PrAl and urethane-isocyanate precursor in the next step was confirmed by the disappearance of the bands of the hydroxyl group (υ -O-H at ca. 3600 cm^−1^) and the isocyanate group (υ -NCO at ca. 2264 cm^−1^) (Figure 6b). According to the FTIR and ^1^H NMR spectra of the monomer mixture, 1,4- and 1,5-disubstituted-1,2,3-triazoles were formed in the AAC reaction at room temperature. Under these conditions, the conversion of ethynyl groups did not exceed 10% over the seven days of storage time (Figure 7).

### 3.3. Structural, Molecular Weight, Hydrodynamic and Thermal Characteristics of Hyperbranched Polyurethane-Triazoles

We compared simulated parameters with experimental data obtained using ^1^H NMR, to validate a developed structural-kinetics model (Table 1).

DB_exp_ was calculated from the amount of linear (L) and dendric (D) units in polymer chains, which in turn was estimated from ^1^H NMR data. The signals with chemical shifts in the range 5.50–4.97 ppm (Figure 7) are from the CH-group of D and L units of polymer chains [19]. In particular, the CH-group (11″) with δ = 5.50 – 5.30 ppm belongs to the D-unit, whereas the CH-group (11′) from L-units has signals in the range of 5.27–4.97 ppm. As can be seen, this signal overlaps with those of the CH_2_-groups (13′ ^1,4^, 13′ ^1,5^) from 1,4- and 1,5-disubstituted 1,2,3-triazoles. Therefore, the amount of CH-group of L-unit can be calculated by subtracting the number of triazoles from the integrated signal in the range 5.27–4.97 ppm. Triazoles has two resolved signals (12′ ^1,4^, 12′ ^1,5^) from 1,4- and 1,5-disubstituted derivatives at 8.20–7.99 ppm and 7.79–7.64 ppm, respectively.

The DB of obtained polymers has a trend of growth with increasing amounts of the *AB*_2_- and *A*_2_-type monomers. On the other hand, DB parameter become lower when the amount of *B*_4_-type monomer increases. The maximum DB value of the polymers was 0.44 (Table 1), which is close to DB in the case of polymerization of a single *AB*_2_-type monomer.

**Figure 8:**
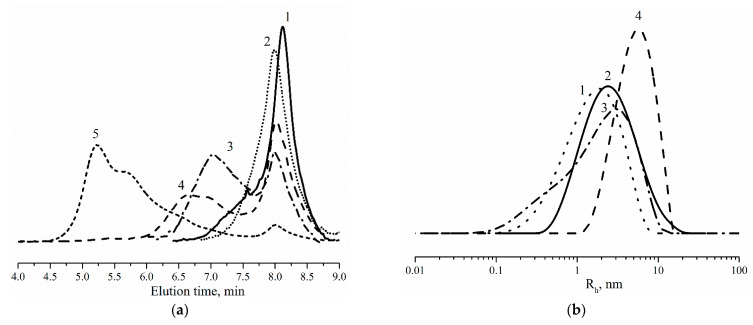
SEC profiles (**a**) and particle distribution (**b**) of hyperbranched polyurethane-triazoles obtained at [NCO]_0_/[OH]_0_ as: 1.50 (1); 1.57 (2); 1.70 (3); 1.81 (4); 1.88 (5).

The molecular weight characteristics presented in Table 1 were found using size-exclusion chromatography (Figure 8). The weight average molecular weight (M_w_^LS^) was found using a light-scattering detector. Figure 8a shows that the molecular weight of the polymer grows with increasing the ratio [NCO]_0_/[OH]_0_. This behavior can be explained by the simultaneous decrease in the amount of *B*_4_-type monomer in the *AB*_2_ + *A*_2_ + *B*_4_ monomer mixture and the increase in the amount of *AB*_2_- and *A*_2_-type monomers.

Multimodal molecular weight distribution is due to the presence in the mixture of two (*AB*_2_ + *B*_4_, for samples 1–3) or three (*AB*_2_ + *A*_2_ + *B*_4_, for samples 4–6) monomers at the same time. Monomer *B*_4_-type act as a termination agent that will define the molecular weight of the formed polymer. In the case of an excess of the *B*_4_-type monomer in the mixture, low-molecular-weight polymers with a low polydispersity index can be obtained. This can be seen on SEC for samples 1 and 2. When decreasing the amount of *B*_4_-type monomer to 0.1–0.2 equivalent with respect to the *AB*_2_-type monomer, high molecular weight hyperbranched polymers are formed that can be observed on SEC as peaks with elution time in the range of 6.0–7.7 min (samples 3 and 4). It should be mentioned that the *A*_2_-type monomer in the mixture of the three monomers *AB*_2_ + *A*_2_ + *B*_4_ acts as a cross-linking agent for macromolecules of branched polymers. This is the reason for the observed increase in the molecular weight of the obtained polymers in the series 3-4-5, as well as a significant increase in polydispersity. As a result, when the concentration of the *A*_2_-type monomer reaches a critical value of 0.03–0.04 equivalent with respect to the *AB*_2_-type monomer (the ratio of the concentrations of *A*_2_- and *B*_4_-type monomers is close to 0.3–0.5, and differs from the previously predicted 0.67, theoretically), the solubility of the resulting polymers in N-methylpyrrolidone becomes extremely poor.

The rheological characteristics of hyperbranched polyurethane-triazoles were studied in the same conditions as molecular weight characteristics. As expected, the viscosity ([η]) of polymers increased with the value of [NCO]_0_/[OH]_0_ and hence the molecular weights of the polymers (Table 1). Based on the viscosity parameter, we calculated the critical overlap concentration of macromolecules in solution that is needed for estimation of the average size of particles. The hydrodynamic radius (R_h_) of particles was calculated using the Stokes–Einstein equation for spherical objects. R_h_ values correlate with the molecular weight of resulting polymers. The average particle size (R_h_^p^) shifts from 1.9 nm to 5.7 nm in a maximum of the size distribution for polymers obtained at [NCO]_0_/[OH]_0_ = 1.50 compared with polymers at [NCO]_0_/[OH]_0_ = 1.88 (Table 1). This enables control of particle size by variation of the ratio of the starting components.

We note that all obtained results are valid for soluble polymers which can be synthesized when the ratio [NCO]_0_/[OH]_0_ does not exceed the critical range of 1.88–1.93. Otherwise, the cross-linking leads to forming a rigid polymer network.

**Figure 9:**
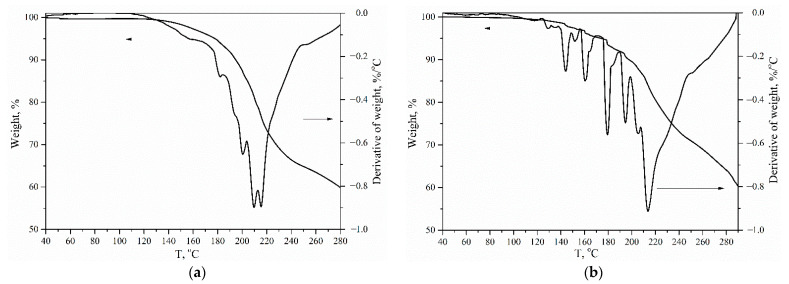
TGA curves of polymers obtained at [NCO]_0_/[OH]_0_ = 1.5 (**a**) and [NCO]_0_/[OH]_0_ = 1.88 (**b**). The measurements were performed under a nitrogen atmosphere, with a heating rate of 2 °C/min.

The thermal properties of the hyperbranched polyurethane-triazoles were investigated using thermal gravimetric analysis (Figure 9). Decomposition temperatures (T_d_) for all obtained polymers were almost the same (230 °C). Since the usual heating rate resulted in multiple explosion effects of polymers, the measurements were performed at a lower heating rate of 2 °C/min. Significant difference can be observed for the destruction kinetics of polymers in the temperature range 150 to 290 °C. The first derivative TGA curve for a polymer obtained at [NCO]_0_/[OH]_0_ of 1.5 exhibited the single maximum evidencing standard decomposition mechanism. At the same time, the derivative TGA curve for the polymer obtained at [NCO]_0_/[OH]_0_ = 1.88 demonstrated multiple peaks that can be attributed to the above-mentioned multiple explosions. Similar effects are typical for azide-containing compounds. However, multiple explosions of obtained polymers might be explained by a high local concentration of azido groups on the periphery of polymers, affecting their thermal stability.

## 4. Conclusions

In this work we performed a complex investigation on the synthesis of hyperbranched polyurethane-triazoles from an *AB*_2_ + *A*_2_ + *B*_4_ azide-acetylene monomers mixture. The proposed one-pot method for the synthesis of hyperbranched polyurethane triazoles consists of three stages. The first step is forming a diazide-isocyanate precursor through a reaction between DAPOL and a lack of asymmetric IPDI. In the second stage, the azide-acetylene monomer mixture is obtained by the reaction of the diazide-isocyanate precursor with PrAl. Finally, the synthesis of hyperbranched polyurethane-triazoles can be carried out through the step-growth polymerization of the *AB*_2_ + *A*_2_ + *B*_4_ monomer mixture using the AAC reaction.

Firstly, the relationships were established between the [NCO]_0_/[OH]_0_ ratio at the stage of diazide-isocyanate precursor formation and the composition of the mixture of *AB*_2_ + *A*_2_ + *B*_4_ monomers formed at the second stage of synthesis. For this, a kinetic model of the reaction of IPDI with DAPOL was developed, considering the differences in the reactivity of the NCO groups of the diisocyanate. Using RPC, it was found that the results of the calculation are in full agreement with the experimental data. We predicted that synthesis of non-cross-linked polyurethane-triazoles without additional purification of the monomer mixture, can be carried out only by [NCO]_0_/[OH]_0_ < 1.95.

Next, a structural kinetic model for the polyaddition of the *AB*_2_ + *A*_2_ + *B*_4_ mixture was developed, considering the possibility of change in activity of the B functional groups. This model allowed the revealing of the relationships between the ratio of [NCO]_0_/[OH]_0_ when synthesizing the diazide-isocyanate precursor and the branching degree of hyperbranched polyurethane-triazoles. Based on calculations, starting conditions were established for the synthesis of the *AB*_2_ + *A*_2_ + *B*_4_ ([NCO]_0_/[OH]_0_ = 1.5–1.93) mixture with a degree of branching from 0.25 to 0.46. Target hyperbranched polyurethane-triazoles were synthesized, and it was shown that the polymer is insoluble in NMP when [NCO]_0_/[OH]_0_ = 1.93. Nevertheless, the maximum DB achieved in the thermo-induced reaction AAC of *AB*_2_ + *A*_2_ + *B*_4_ (if [NCO]_0_/[OH]_0_ = 1.88) was 0.44 (experimental) and 0.45 (calculated). Based on the results obtained from the structural-kinetic model and previously reported data, it would be expected that the value of DB could be increased when the CuAAC reaction was performed.

According to the results of the investigation of hyperbranched polyurethane-triazoles by NMR spectroscopy, FTIR, SEC, DLS, and viscometry, it was found that the degree of branching, molecular weight, hydrodynamic radius and intrinsic viscosity of the obtained hyperbranched polymers are controlled by the ratio [NCO]_0_/[OH]_0_ at the first stage of synthesis. We believe that these promising hyperbranched polyurethane-triazoles containing azide side groups can be modified in the reactions with terminal alkynes, which opens numerous opportunities for obtaining novel functional polymers for a wide range of applications.

## Figures and Tables

**Table 1:** Structural parameters, molecular weights, hydrodynamic and thermal characteristics of hyperbranched polyurethane-triazoles.

[NCO]_0_/[OH]_0_	[*AB*_2_]:[*A*_2_]:[*B*_4_]	DB_exp_ (DB_calc_)	M_w_^LS^	[η]·10^2^ (dl/g)	R_h_^p^ (nm)	T_g_ (°C)	T_d_ (°C)
1.50	1:0:0.50	0.21 (0.25)	10,100	3.5	1.9	40	230
1.57	1:0:0.35	0.28 (0.29)	10,800	4.0	2.4	53	228
1.70	1:0:0.20	0.33 (0.35)	15,900	5.6	3.0	69	231
1.81	1:0.008:0.13	0.39 (0.40)	28,600	7.8	5.7	73	232
1.88	1:0.025:0.097	0.44 (0.45)	174,700	-	-	86	230
1.93	1:0.042:0.083	Cross-linking				90	232

Here [NCO]_0_/[OH]_0_—initial ratio in reaction DAPOL with IPDI; [*AB*_2_]:[*A*_2_]:[*B*_4_]—the molar ratio of monomers in *AB*_2_ + *A*_2_ + *B*_4_ monomer mixture; DB_exp_, DB_calc_—degree of branching of synthesized polymers using experimental and calculation data, respectively; M_w_^LS^—weight average molecular weight obtained using light-scattering detector; [η]—intrinsic viscosity; R_h_^p^—average hydrodynamic radius; T_g_—glass transition temperature; T_d_—decomposition temperature.

## Data Availability

Not applicable.

## Supplementary Materials

The following supporting information can be downloaded at: https://www.mdpi.com/article/10.3390/polym14214514/s1, Figure S1: ^1^H NMR spectra in DMSO-d6 of *AB*_2_ + *A*_2_ + *B*_4_ monomers mixture synthesized at ratio [NCO]_0_/[OH]_0_ = 1.7
