# Supplementary material for: One-Pot Synthesis of Hyperbranched Polyurethane-Triazoles with Controlled Structural, Molecular Weight and Hydrodynamic Characteristics
Source: Polymers (Basel). 2022 Oct 25;14(21):4514. doi: 10.3390/polym14214514 (PMC9656031; doi:10.3390/polym14214514)
Supplement: Supplementary file 1 [file polymers-14-04514-s001.zip › polymers-1981120-supplementary.pdf]

polymers

MDPI

Supplementary Materials

One-Pot Synthesis of Hyperbranched Polyurethane-Triazoles with Controlled Structural, Molecular Weight and Hydrodynamic Characteristics

Sergei V. Karpov [1,*], Artem Iakunkov [2], Alexander V. Akkuratov [1], Artem O. Petrov [1], Eugenia O. Perepelitsina [1], Georgiy V. Malkov [1], Elmira R. Badamshina [1]

1 Department of Polymers and Composite Materials, Federal Research Center of Problems of Chemical Physics and Medicinal Chemistry of Russian Academy of Sciences, Chernogolovka, 142432, Russia
2 Department of Fibre and Polymer Technology, KTH Royal Institute of Technology, Stockholm, SE-100 44, Sweden
* Correspondence: svkarpov@icp.ac.ru

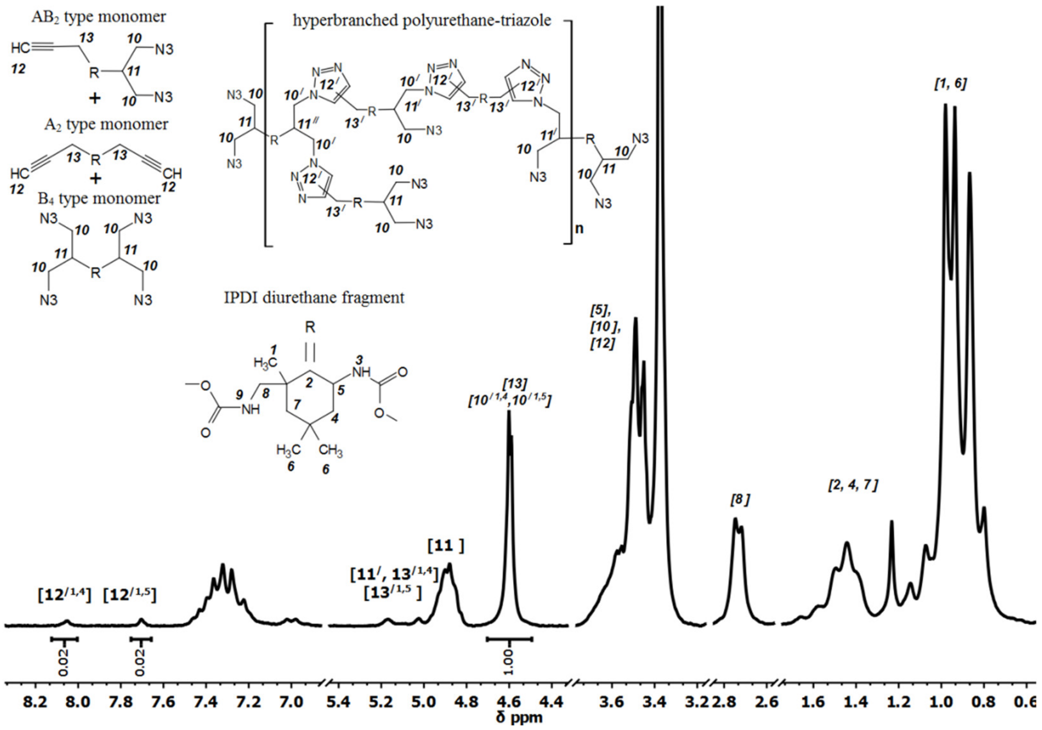

Figure S1. ^{1}H NMR spectra in DMSO-d6 of AB_2+A_2+B_4 monomers mixture synthesized at ratio $[NCO]_0/[OH]_0 = 1.7$.

Figure S1 shows typical ^{1}H NMR spectrum of AB_2+A_2+B_4 mixture, obtained by ratio $[NCO]_0/[OH]_0 = 1.7$ after storage for seven days.

Due to overlap of signals, calculation of ethinyl groups conversion is difficult. It can be carried out assuming the next:

The signals by 4.71 – 4.51 ppm (13) can be assigned to CH_2 group by non-reacted ethinyl groups of AB_2 and A_2 type monomers. The strong signal (13) overlap the signals ($10'^{1,4}$, $10'^{1,5}$) of CH_2 group by N atom in formed 1,4- and 1,5-triazoles (see ^{1}H NMR of polymer, **Figure 7**). From synthesis it is obviously that integral intensity of signals ($10'^{1,4}$, $10'^{1,5}$) should be equal to integral intensity of signals ($13'^{1,4}$, $13'^{1,5}$) of CH_2 by ethinyl groups reacted in AAC. Thus, the starting amount of ethinyl group will be proportional

to half of total integral intensity of signal with chemical shift 4.71 – 4.51 ppm (1 proton of CH group (12) correspond to 2 protons of CH_2 groups (13))

Protons of CH groups in triazoles has two clear resolved signals ($12'^{1,4}$, $12'^{1,5}$) from 1,4- and 1,5-disubstituted derivatives at 8.20 – 7.99 ppm and 7.79 – 7.64 ppm, respectively. The amount of reacted ethinyl groups will be proportional to sum of integral intensities of signals by 8.20 – 7.99 ppm and 7.79 – 7.64 ppm.

Based on this, we calculated the conversion of ethinyl groups in mixture of AB_2+A_2+B_4 monomers is about 8%.
